# Composition of salivary microbiota in elderly subjects

**DOI:** 10.1038/s41598-017-18677-0

**Published:** 2018-01-11

**Authors:** Taiji Ogawa, Yujiro Hirose, Mariko Honda-Ogawa, Minami Sugimoto, Satoshi Sasaki, Masahito Kibi, Shigetada Kawabata, Kazunori Ikebe, Yoshinobu Maeda

**Affiliations:** 1 Department of Prosthodontics, Gerodontology and Oral Rehabilitation, Osaka University Graduate School of Dentistry, 1-8, Yamadaoka, Suita, Osaka, 5650871 Japan; 2 Department of Oral and Molecular Microbiology, Osaka University Graduate School of Dentistry, 1-8, Yamadaoka, Suita, Osaka, 565-0871 Japan; 3 Department of Social and Preventive Epidemiology, School of Public Health, The University of Tokyo, 7-3-1 Hongo, Bunkyo, Tokyo, 1130033 Japan

## Abstract

Frailty is gaining attention worldwide with the aging of society. Despite the potential lethality and multiple signs and symptoms in affected individuals, preclinical detection of early manifestations leading to frailty syndrome have not been established. We speculated that the composition of the oral microbiota is associated with general frailty, as well as a relationship between gut microbiota and general health condition. In the present study, we investigated the salivary microbiota composition in samples from healthy and frail elderly individuals using 16S rRNA sequencing analysis for characterization. We found a significant difference in diversity between elderly individuals living in a nursing home (EN) and healthy control (HC) subjects, as well as in the microbiota composition at the phyla level. A supervised orthogonal partial least squared discriminant analysis (OPLS-DA) revealed a significant difference in clear classification trend between the EN and HC groups, with all observations falling within the Hotellings T^2^ (0.95) ellipse, with model fitness parameters of *R* ^2^(cum) = 0.937 and *Q* ^2^(cum) = 0.888, respectively. In addition, the score plots by unsupervised principal component analysis (PCA) showed a clear classification trend in both groups. Our findings suggest that general frailty is associated with oral microbiota composition and formation.

## Introduction

Frailty is gaining attention worldwide along with the general aging of society. It is theoretically defined as a clinically recognizable state of increased vulnerability resulting from age-associated declines in reserve and function across multiple physiological and psychological systems, with the result being a comprised ability to cope with daily or acute stressors, as well as socio-economic difficulties^1,2^. Despite the potential lethality of frailty and its multiple signs and symptoms, including functional disability, various associated diseases, physical and cognitive impairments, psychosocial risk factors, and geriatric syndromes, such as falls, delirium, and urinary incontinence^3^, methods for preclinical detection of early manifestations leading to the frailty syndrome have not been established.

Recent developments in techniques for 16S ribosomal RNA sequencing have enabled comprehensive analysis of commensal microbiota in examined specimens, with more than 700 bacterial species detected in the oral cavity^4^. The varieties of organisms form unique communities, though the microbial composition is thought to remain stable within individuals^5^. The relationship between gut microbiota and aging has been suggested to have effects on the health of older adults^6^, while Jackson *et al*. reported a negative association between frailty and gut microbiota diversity^7^. Presently, gut microbiota is recognized as a key factor for support of intestinal homeostasis and health. Additionally, dysbiosis of gut microbiota has been suggested to be induced by oral administration of pathogenic bacteria in an animal model^8^. In addition, dietary sources are suggested to impact microbiota ecology^9^.

We speculated that oral microbiota composition is associated with general frailty, in addition to the relationship between gut microbiota and general health condition. In the present study, we investigated salivary microbiota using 16S rRNA sequencing analysis in samples obtained from healthy and frail elderly subjects, and aimed to define and characterize the salivary microbiota in both groups, along with aspects of nutritional intake.

## Results

### Profiling of oral microbiota in elderly subjects with general frailty

**Figure 1:**
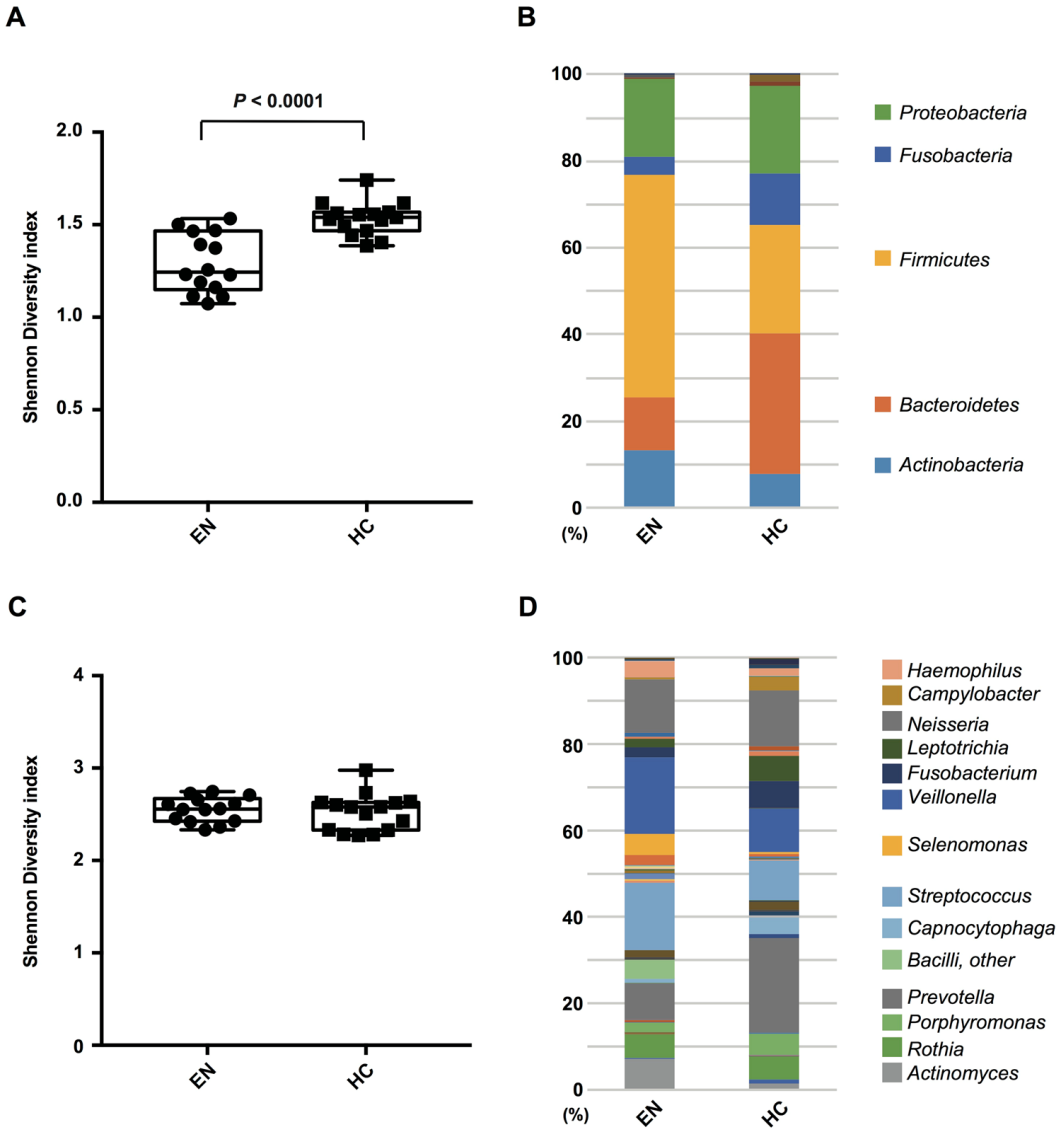
Salivary microbiota diversity and community. (**A**) Index (Shannon, *y*-axis) of phyla diversity. Data are shown as a box-whisker plot (minimum, lower quartile, median, upper quartile, maximum). Statistical significance was calculated using Mann-Whitney’s *U* test (*P* < 0.0001). (**B**) Bar chart showing average bacterial profile of phyla. (**C**) Index (Shannon, *y*-axis) of genera diversity. (**D**) Bar chart showing average bacterial profile of genera. EN, elderly subjects living in nursing home; HC, healthy control subjects.

**Figure 2:**
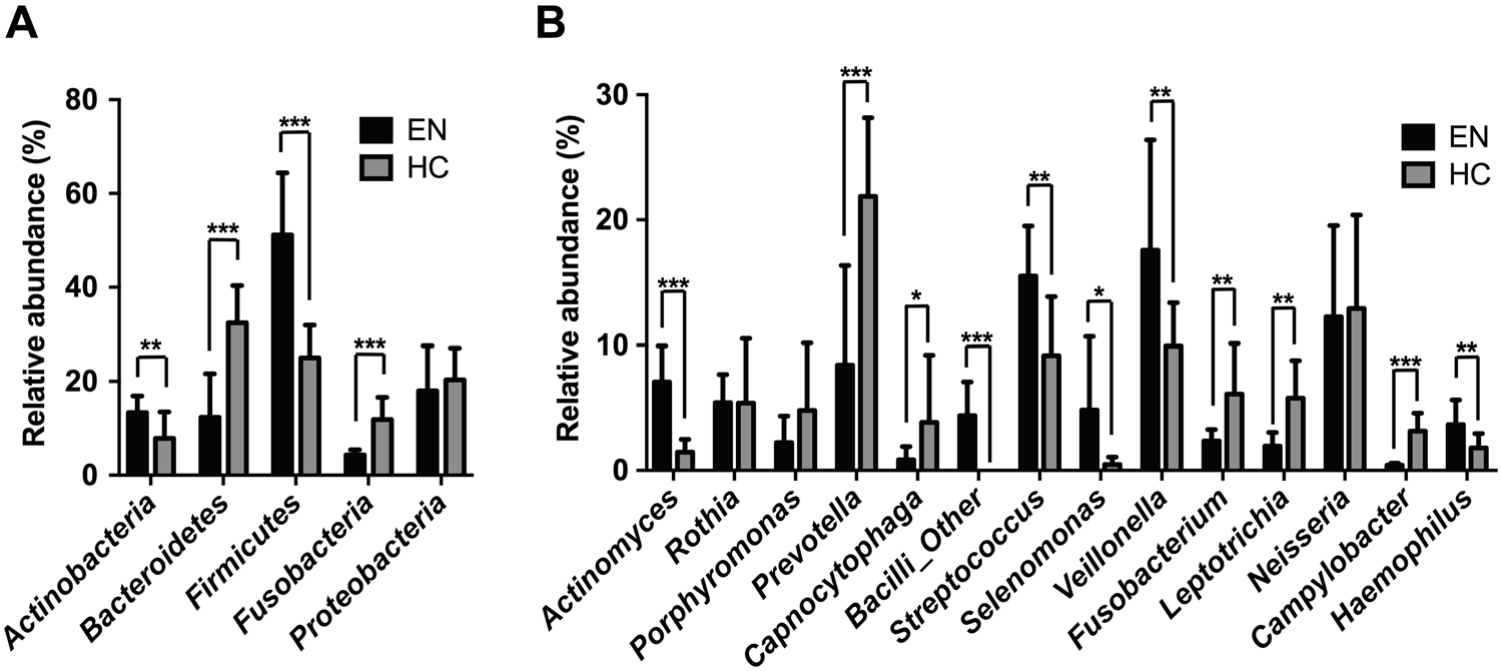
Salivary microbiota composition in different taxa. Bar charts showing average bacterial composition of different taxa of the (**A**) phylum and (**B**) genus in each group (black bars: EN, grey bars: HC). *P < 0.05, **P < 0.01 ***P < 0.001.

Salivary samples were analyzed using metagenomic 16S rRNA pyro-sequencing at each phylogenetic level from phylum to genus. Our results showed that the EN and (HC) groups had distinct patterns of numerically dominant bacterial taxa. The basic profiles of the subjects are listed in Table 1. At the phyla level, the Shannon Diversity index *H* value for the EN samples was significantly lower than that for the HC samples (Fig. 1A). The significant difference in OTUs between the groups included a lower relative abundance of *Bacteroidetes* (*P* < 0.01) and *Fusobacteria* (*P* < 0.001), and higher relative abundance of *Actinobacteria* (*P* < 0.001) and *Firmicutes* (*P* < 0.001) in the EN group (Figs 1B and 2A).

**Table 1:** Basic profile of the participants.

	Elderly in nursing home (EN)	Independent living healthy control (HC)
Number of participants	15	16
Nursing facility A (total participants)	3 (9)	—
Nursing facility B (total participants)	2 (6)	—
Nursing facility C (total participants)	10 (35)	—
Male/Female	3/12	9/7
Age [yeas] (mean ± s.d.)	84.2 ± 7.7	87.0 ± 4.6
BMI [kg/m^2^] (mean ± s.d.)	22.1 ± 3.1	21.8 ± 2.6
Denture wearers	12	13
Edentulous participants	3	4
Dentate participants	12	12
Number of teeth (median, range)	11, 3–30	10, 0–28
Number of Decayed teeth (median, range)	0, 0–4	0, 0–0
Number of Missing teeth (median, range)	17, 2–25	18, 0–28
Number of Filled teeth (median, range)	6, 1–14	4, 0–17

At the genera level, we found no significant difference between the groups in regard to Shannon Diversity index (Fig. 1C). On the other hand, the EN group showed a higher composition of *Actinomyces* (*P* < 0.001), *Streptococcus* (*P* < 0.001), *Selenomonas* (*P* = 0.0123), *Veillonella* (*P* < 0.01), and *Haemophilus* (*P* < 0.01), and lower composition of *Prevotella* (*P* < 0.001), *Capnocytophaga* (*P* < 0.05), *Fusobacterium* (*P* = 0.0022), *Leptotrichia* (*P* < 0.001), and *Campylobacter* (*P* < 0.001) as compared to the composition in the HC group (Figs 1D and 2B).

### Clustering of oral microbiota samples from elderly subjects

**Figure 3:**
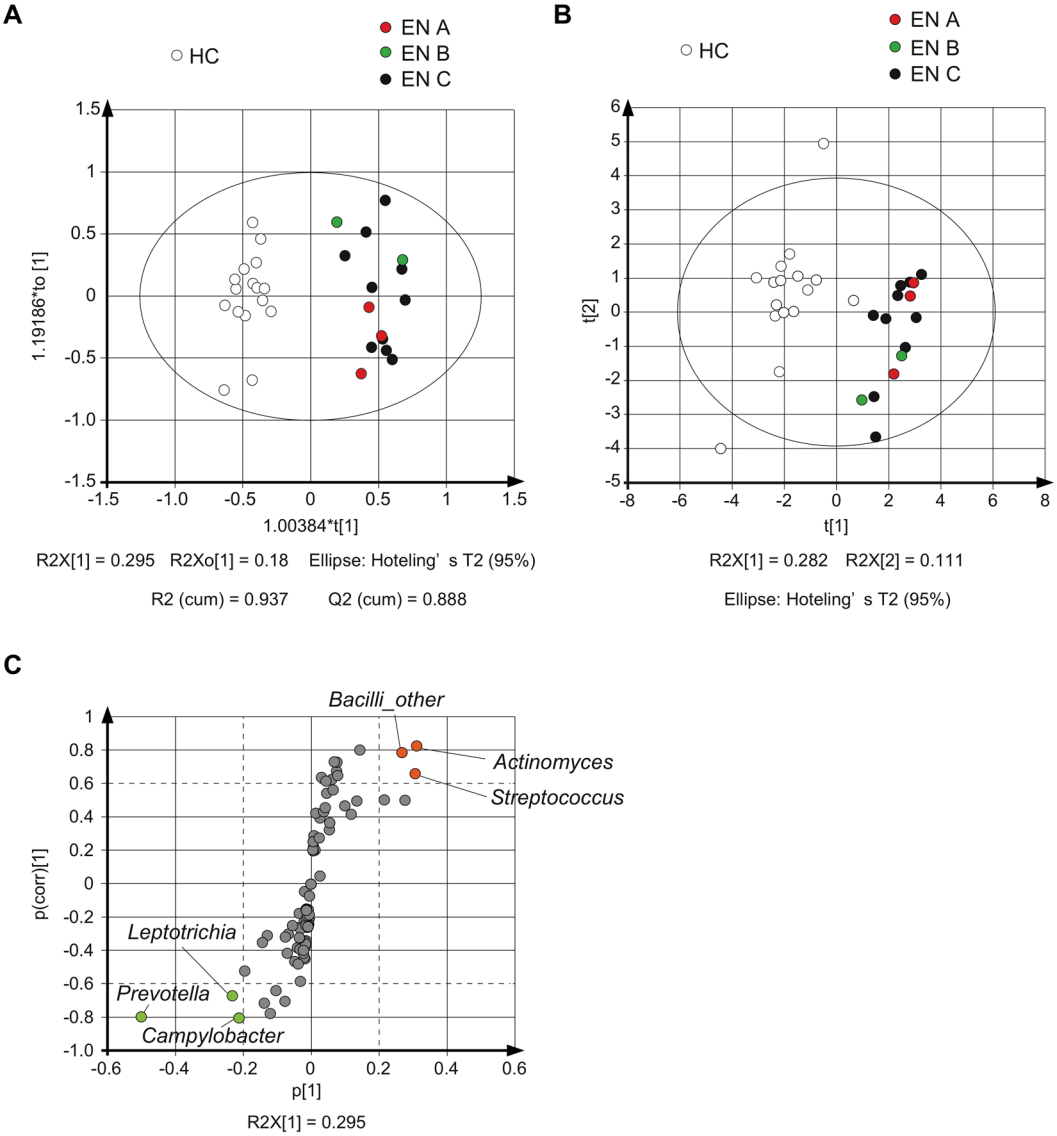
Visualization of components related to differences in oral microbial composition between EN and HC groups. (**A**) OPLS-DA and (**B**) PCA score plots summarizing features of genus found in salivary flora in the HC and EN groups, which are highlighted by colored (EN A; red, EN B; green, EN C; black) and white circles, respectively. (**C**) S-plots from OPLS-DA. Genera showing differences were *Actinomyces*, *Streptococcus*, and *Bacilli*_other, which had a higher relative abundance in EN (red), and *Prevotella*, *Leptotrichia*, and *Campylobacter*, which had a lower relative abundance in EN (green).

Application of a traditional univariate statistical method (e.g., Student’s *t*-test) to these complex datasets may result in a high number of false positives, while the predominant approach of *P*-value correction performed to account for these high false positive rates is associated with a significant loss in statistical power. On the other hand, a multivariate statistical analysis approach is a powerful tool for integration and interpretation of such datasets towards sub-phenotypes^10^. To broadly evaluate the differences between the EN and HC groups, supervised orthogonal partial least squared discriminant analysis (OPLS-DA) was performed. The score plots demonstrated a clear classification trend in both groups, with all observations falling within Hotellings T^2^ (0.95) ellipse, and the model fitness parameters were *R* ^2^(cum) = 0.937 and *Q* ^2^(cum) = 0.888, respectively (Fig. 3A). In addition, the score plots by unsupervised principal component analysis (PCA) showed a clear classification trend in both groups as shown in Fig. 3B. While, we found no difference among the three nursing homes by OPLS-DA and PCA analyses.

VIP is generally used as a metric to summarize the importance of each variable for driving model construction. For stringent selection of factors related to the differences between the groups, we focused on genera with VIP values higher than 1.0 (Table 2). From the S-plot of OPLS-DA, genera mainly responsible for discrimination of the EN and HC group could be extracted (Fig. 3C). In an S-plot, the distance from the *y*-axis indicates the correlation coefficient between two groups^10^. Thus, we considered that a p(corr) value > 0.6 represented a significant difference (Table 3). Our analysis revealed that genera contributed to the difference, including *Actinomyces*, *Streptococcus*, and *Bacilli_*other, which had a higher relative abundance, in addition to the genera with a lower relative abundance in EN, such as *Prevotella*, *Leptotrichia*, and *Campylobacter* (Fig. 3C). Those genera were also related to high VIP (Table 2) and significant differences (*t*-test) between the groups (Fig. 2). There is no consensus regarding what p(corr) cutoff level represents significance, though an absolute value of >0.4–0.5 is often used^10^. Based on the above, our findings indicated that oral samples obtained from the present nursing home residents showed greater levels of *Selenomonas*, *Veillonella*, and *Haemophilus*, and lower levels of *Fusobacterium*.

**Table 2:** VIP of OPLS-DA.

Phylum	Class	Order	Family	Genus	VIP
*Bacteroidetes*	*Bacteroidia*	*Bacteroidales*	*Prevotellaceae*	*Prevotella*	6.3938
*Firmicutes*	*Clostridia*	*Clostridiales*	*Veillonellaceae*	*Veillonella*	4.4453
*Actinobacteria*	*Actinobacteria*	*Actinomycetales*	*Actinomycetaceae*	*Actinomyces*	3.9698
*Firmicutes*	*Bacilli*	*Lactobacillales*	*Streptococcaceae*	*Streptococcus*	3.9400
*Firmicutes*	*Bacilli*	*Other*	*Other*	*Other*	3.3435
*Proteobacteria*	*Betaproteobacteria*	*Neisseriales*	*Neisseriaceae*	*Neisseria*	3.1566
*Fusobacteria*	*Fusobacteriia*	*Fusobacteriales*	*Leptotrichiaceae*	*Leptotrichia*	2.9550
*Firmicutes*	*Clostridia*	*Clostridiales*	*Veillonellaceae*	*Selenomonas*	2.9332
*Fusobacteria*	*Fusobacteriia*	*Fusobacteriales*	*Fusobacteriaceae*	*Fusobacterium*	2.7598
*Proteobacteria*	*Epsilonproteobacteria*	*Campylobacterales*	*Campylobacteraceae*	*Campylobacter*	2.6107
*Proteobacteria*	*Gammaproteobacteria*	*Pasteurellales*	*Pasteurellaceae*	*Haemophilus*	2.0511
*Bacteroidetes*	*Bacteroidia*	*Bacteroidales*	*Porphyromonadaceae*	*Porphyromonas*	2.0399
*Firmicutes*	*Clostridia*	*Clostridiales*	*Veillonellaceae*	*Megasphaera*	1.9114
*Actinobacteria*	*Actinobacteria*	*Actinomycetales*	*Micrococcaceae*	*Rothia*	1.8013
*Firmicutes*	*Clostridia*	*Clostridiales*	*Eubacteriaceae*	*Pseudoramibacter_Eubacterium*	1.79312
*Bacteroidetes*	*Flavobacteriia*	*Flavobacteriales*	*Flavobacteriaceae*	*Capnocytophaga*	1.7714
*TM7*	*TM7–3*	Other	Other	Other	1.7026
*Firmicutes*	*Bacilli*	*Gemellales*	*Gemellaceae*	Other	1.4795
*Bacteroidetes*	*Bacteroidia*	*Bacteroidales*	[*Paraprevotellaceae*]	[*Prevotella*]	1.2790
*Proteobacteria*	*Betaproteobacteria*	*Neisseriales*	*Neisseriaceae*	*Eikenella*	1.2514

**Table 3:** p(corr) of S plot.

Phylum	Class	Order	Family	Genus	p(corr)	p[1]
*Actinobacteria*	*Actinobacteria*	*Actinomycetales*	*Actinomycetaceae*	*Actinomyces*	0.821563	0.311517
*Actinobacteria*	*Actinobacteria*	*Actinomycetales*	*Corynebacteriaceae*	*Corynebacterium*	−0.412454	−0.069369
*Actinobacteria*	*Actinobacteria*	*Actinomycetales*	*Propionibacteriaceae*	Other	−0.444868	−0.017355
*Actinobacteria*	*Actinobacteria*	*Bifidobacteriales*	*Bifidobacteriaceae*	*Scardovia*	−0.437573	−0.019687
*Actinobacteria*	*Coriobacteriia*	*Coriobacteriales*	*Coriobacteriaceae*	*Atopobium*	0.622804	0.061248
*Actinobacteria*	*Coriobacteriia*	*Coriobacteriales*	*Coriobacteriaceae*	*Olsenella*	0.422136	0.014765
*Bacteroidetes*	*Bacteroidia*	*Bacteroidales*	*Porphyromonadaceae*	*Tannerella*	−0.460832	−0.047110
*Bacteroidetes*	*Bacteroidia*	*Bacteroidales*	*Prevotellaceae*	*Prevotella*	−0.791755	−0.499932
*Bacteroidetes*	*Bacteroidia*	*Bacteroidales*	[*Paraprevotellaceae*]	[*Prevotella*]	−0.634343	−0.102649
*Bacteroidetes*	*Flavobacteriia*	*Flavobacteriales*	*Flavobacteriaceae*	Other	0.430256	0.038407
*Bacteroidetes*	*Flavobacteriia*	*Flavobacteriales*	[*Weeksellaceae*]	Other	−0.699069	−0.076483
*Firmicutes*	*Bacilli*	Other	Other	Other	0.784003	0.268678
*Firmicutes*	*Bacilli*	*Gemellales*	*Gemellaceae*	Other	−0.771758	−0.119417
*Firmicutes*	*Bacilli*	*Lactobacillales*	*Streptococcaceae*	*Streptococcus*	0.657308	0.307290
*Firmicutes*	*Clostridia*	*Clostridiales*	Other	Other	0.672813	0.075368
*Firmicutes*	*Clostridia*	*Clostridiales*	*Clostridiaceae*	02d06	0.726618	0.077156
*Firmicutes*	*Clostridia*	*Clostridiales*	*Eubacteriaceae*	*Pseudoramibacter_Eubacterium*	0.797483	0.144909
*Firmicutes*	*Clostridia*	*Clostridiales*	*Lachnospiraceae*	*Lachnoanaerobaculum*	0.645967	0.079312
*Firmicutes*	*Clostridia*	*Clostridiales*	*Lachnospiraceae*	*Oribacterium*	0.541669	0.047280
*Firmicutes*	*Clostridia*	*Clostridiales*	*Peptococcaceae*	*Peptococcus*	0.635500	0.0318019
*Firmicutes*	*Clostridia*	*Clostridiales*	*Peptostreptococcaceae*	*Filifactor*	0.613740	0.0442592
*Firmicutes*	*Clostridia*	*Clostridiales*	*Peptostreptococcaceae*	*Peptostreptococcus*	0.727684	0.0686493
*Firmicutes*	*Clostridia*	*Clostridiales*	*Veillonellaceae*	*Acidaminococcus*	0.454836	0.0424878
*Firmicutes*	*Clostridia*	*Clostridiales*	*Veillonellaceae*	*Megasphaera*	0.414778	0.118925
*Firmicutes*	*Clostridia*	*Clostridiales*	*Veillonellaceae*	*Selenomonas*	0.500736	0.216413
*Firmicutes*	*Clostridia*	*Clostridiales*	*Veillonellaceae*	*Veillonella*	0.500022	0.277569
*Firmicutes*	*Clostridia*	*Clostridiales*	[*Mogibacteriaceae*]	Other	−0.412028	−0.0218757
*Firmicutes*	*Erysipelotrichi*	*Erysipelotrichales*	*Erysipelotrichaceae*	*Bulleidia*	−0.579467	−0.0301981
*Fusobacteria*	*Fusobacteriia*	*Fusobacteriales*	*Fusobacteriaceae*	*Fusobacterium*	−0.517298	−0.194807
*Fusobacteria*	*Fusobacteriia*	*Fusobacteriales*	*Leptotrichiaceae*	*Leptotrichia*	−0.664764	−0.231179
*Proteobacteria*	*Betaproteobacteria*	*Neisseriales*	*Neisseriaceae*	*Eikenella*	0.466866	0.100435
*Proteobacteria*	*Epsilonproteobacteria*	*Campylobacterales*	*Campylobacteraceae*	*Campylobacter*	−0.797445	−0.21117

## Discussion

The present study identified bacterial genera in salivary samples that were found to contribute to differences between nursing home residents and community dwelling older adults, based on univariate and multivariate statistical analyses. In samples from the nursing home residents group, *Actinomyces*, *Streptococcus*, *Bacilli*, *Selenomonas*, *Veillonella*, and *Haemophilus* showed higher relative abundance, while *Prevotella*, *Leptotrichia*, *Campylobacter*, and *Fusobacterium* had a lower relative abundance. According to Takeshita *et al*., the dominant genera in their subjects fed orally, such as *Streptococcus* and *Veillonella*, were present in much lower proportions in tube-fed subjects^11^. On the other hand, *Prevotella* and *Veillonella* were commonly dominant in patients with inflammatory bowel disease^12^ and periodontal disease^13^. These findings may indicate the need for elucidation of the linkage between general frailty and oral dysbiosis.

The present participants living in nursing homes received adequate and sufficient oral hygienic care by their caregivers. Nevertheless, the phyla level diversity in the nursing home residents was significantly lower than that in the independent living HC group. Interestingly, loss of intestinal core microbiota diversity has also been reported to be associated with increased frailty^6^. Together, these findings suggest that microbiota diversity is a key issue in regard to local and general health conditions. Nakajima and colleagues demonstrated that oral administration of pathogenic bacteria induced dysbiosis of gut microbiota and subsequent dissemination of intestinal bacteria to the liver in an animal model^8^. They also found that oral administration of *Porphyromonas gingivalis* induced higher mRNA expressions of pro-inflammatory cytokines such as IL-6 and TNF-α in the intestine. Another group supported those findings and showed that oral administration of a bacterial mixture affected gut microbiota^14^. Nevertheless, it is not clear whether oral dysbiosis affects general frailty or if general frailty has effects on oral dysbiosis, thus further study is needed to investigate their association.

The primary initial colonizers in the oral cavity are *Streptococcus* and some *Actinomyces* species, while early colonizing *Veillonella* has been shown to co-aggregate with streptococci and *Actinomyces* ^15^. In the present study, we found that *Streptococcus*, *Veillonella*, and *Actinomyces* were present at higher levels in the composition of salivary microbiota obtained from the nursing home residents. In contrast, *Prevotella*, *Leptotrichia*, *Campylobacter*, and *Fusobacterium*, Gram-negative bacilli, are known to be pathogens causing aspiration pneumonia^16^. Yoneyama *et al*. reported that oral care for elderly residents in nursing homes was effective to reduce pneumonia occurrence, as well as febrile days and death from pneumonia^17^. Also, activities of daily living and cognitive functions showed a tendency to improve with oral care. Residents of nursing homes generally receive relatively sufficient and appropriate oral hygienic care from their caregivers according to professional instructions from dentists and dental hygienists. Previous findings indicate that appropriate oral care is effective to reduce harbored pneumonic bacteria. Nevertheless, *Streptococcus* and *Haemophilus* (e.g., *Streptococcus pneumoniae*, *Haemophilus influenza*) also cause aspiration pneumonia^16^. In saliva, low GC gram-positive bacteria (*Firmicutes*) are known to be the major phyla present as compared to other oral sites^18^. Therefore, it is important to include other sites such as periodontal pockets that have a different environment from saliva when discussing aspiration pneumonia.

Nutrient intakes are recognized as a powerful factor to determine microbial mechanisms and key metabolites that shape the composition of the human gut microbiota^9^. Although the nutrient intakes could not simply be compared because the different dietary assessment methods were used between EN group and HC group, non-energy providing nutrient intakes were tend to be lower among EN group than those among HC group (Supplementary Information and Table S2). Lower intake of such nutrients might influence to the gut microbiota among EN group.

For example, the present nursing home residents ingested relatively lower amount of cobalamin than the HC group did, which suggests that dietary cobalamin intake influences gut microbiota as well as oral microbiota composition. A relationship between cobalamin metabolism and gut microbiota has been suggested^19,20^. Cobalamin is thought to be critical for humans and their gut microbiota, though microbes in the gut are unlikely to be a significant source.

In addition, n-6 PUFAs are known to be predominant in the diets of Western populations^21^, while Japanese individuals habitually eat more fish and shellfish, which are rich in n-3 PUFAs including docosahexaenoic acid^22^. A positive correlation of the percentage of n-3 fatty acids in blood 20- and 22-carbon PUFAs with daily menu balance values determined by the balance of n-6 and n-3 fatty acids in food has been reported^23^. The n-6/n-3 balance as a total amount in our subjects seemed well controlled in both groups, though the EN group ingested relatively less of some of the n-6 PUFAs examined. This result may also provide a hint to understand the link between food ingestion and microbiota.

In a previous study, an age-related decrease in T cell function and compensatory increase in the function of antigen-presenting cells was found in healthy elderly subjects with maintained immune response. On the other hand, decreases in the functions of both T cells and antigen-presenting cells increased the susceptibility of frail elderly individuals to infections^24^. Those findings may have been confirmed by our result showing that the microbial composition was categorized differently between the nursing home residents and independent living elderly control subjects. Immunologic differences between healthy and frail elderly may be the result of important changes in dendritic cell function and regulation influenced by age and/or environment. Intestinal dendritic cells are loaded with commensal bacteria and have been shown to promote generation of protective IgA, and may also be required, in addition to lung dendritic cells, for generation of mucosal IgA in elderly humans^25,26^. This process allows dendritic cells to selectively induce IgA, which helps protect against mucosal penetration by commensals. Immune responses to commensal bacteria are locally induced without potential damaged to systemic immune responses^27^. Host immunity may be responsible for alteration of microbiota in individuals with general frailty.

This study has several limitations. First, the periodontal status of the subjects was not evaluated because of insufficient effort for such an investigation in the nursing home residents, though chronic periodontitis is known to be a factor associated with the composition of subgingival microbiota^28^. In the same oral cavity, the environment can differ based on location, including buccal, vestibule, tongue, palate, tonsil, tooth surface, gingival, and other tissues, thus bacterial composition is site specific^4^. We examined saliva samples in this investigation. However, the relationship between microbiota in saliva and subgingival sites is complex.

In addition, we have recognized that the small sample size is another limitation of our study, with lack of power to support the present findings. Moreover, we lost 35 of 50 potential samples from EN, because we only utilized the subjects who were able to provide sufficient quantities of saliva for microbial analysis in the current protocol. The selection method may cause another bias for the result within EN samples, such that participants with less frailty are more likely to be included in the analysis because salivary flow is known to reduce with aging. Although there are reports of links between aging and microbiota, it is difficult to determine the precise associations because of several changes that occur with aging^29^, including living conditions, such as visits to day hospitals or a permanent move to a long-term care facility. It is important to note that previous studies have shown that the microbiota of individuals living within the same household tend to be similar^30^. Therefore, the varying backgrounds of the present participants may have affected the results. Nevertheless, several studies have suggested associations between microbiota and general frailty^7,29,31^. To elucidate such a linkage, future studies that use both biological and epidemiological approaches across host species, age, and race, as well as a considerable number of other factors are necessary.

In conclusion, we observed different salivary microbiota clusters between elderly individuals living in a nursing home and those living independently who were nearly the same age using multivariate statistical analysis. Our results suggest that general frailty is one of the factors associated with oral microbiota formation and composition.

## Materials and Methods

### Study population

A total of 50 participants residing at 3 different nursing homes in Osaka, Japan were recruited for this study. In order to use the services covered by Japanese long-term care insurance, the applicant need to be certificated based on written opinion by medical doctor that unable to live at home because of general frailty (http://www.mhlw.go.jp/english/policy/care-welfare/care-welfare-elderly/dl/ltcisj_e.pdf). From those, 15 elderly subjects (elderly in nursing home; EN group, 68–101 years old) who were able to provide sufficient quantities (1 ml or more) of whole saliva for microbial analysis were selected. We could obtain insufficient amount of salivary sample from the 35 subjects. The diseases and disorders were listed in Table S1. In addition, 16 healthy independent living older adults who participated in the SONIC Study^32^ (79–94 years old) were enrolled as controls (healthy control; HC group). These 16 subjects were selected because they participated in a random day of salivary examination, who provided sufficient quantities of whole saliva. Subjects who had been administrated antimicrobial agents within the previous 3 months were excluded from analysis. The study protocol was approved by the Institutional Review Board of Osaka University Graduate School of Dentistry (H22-E9) and performed in accordance with approved guidelines. All subjects provided written informed consent prior to participation.

### Sample collection

During the period from November 2014 to January 2016, saliva samples were collected into disposable cups after the subjects had refrained from all intake of food and drink, smoking, and use of toothpaste for at least 2 hours. The samples were stored on ice during the collection procedure and immediately processed for genomic DNA extraction. We excluded participants with insufficient saliva collection (less than 1.0 ml of whole saliva) or an insufficient amount of extracted genomic DNA.

### DNA extraction

Genomic DNA was extracted from each saliva sample using a Power Soil DNA Isolation Kit (MO BIO Laboratories, Carlsbad, CA, USA). DNA purification was performed with phenol-chloroform isoamyl alcohol (25:24:1, v/v) using DNA extraction and ethanol precipitation methods.

### 16S rRNA library preparation and DNA sequencing

A 16S ribosomal RNA library was constructed using a TruSeq DNA sample preparation kit (Illumina, San Diego, CA, USA) and quantified with a Qubit^®^ dsDNA HS Assay kit (Thermo Fisher Scientific Inc., Waltham, MA, USA). V1-2 hypervariable regions of bacterial 16S rRNA genes were amplified using custom barcode primers (Fw: 5′-AGRGTTTGATCMTGGCTCAG-3′; Rv: 5′-TGCTGCCTCCCGTAGGAGT-3′) and sequenced with paired-end 250-bp reads with an Illumina MiSeq.

### Quality filtering

A FASTX-Toolkit (version 0.013) was employed to process the raw sequencing data, using the following quality criteria. [1] The minimum acceptable Phred quality score for the sequences was 20, with a score of ≥20 noted in more that 70% of the sequence bases. [2] After quality trimming from the sequence tail, sequences over 280 bp were retained and they also had an acceptable Phred quality score of 20. [3] Both forward and reverse sequencing (merged sequencing reads from both primers) that met the first and second requirements were retained for subsequent analysis.

### Taxonomy assignment and sequence analyses

MAFFT was used to align the operational taxonomic units (OTUs) with the Greengenes (gg_13_8) database. A standard of 97% similarity with the database was applied and sequencing reads that did not match the database were removed.

### Statistical data analysis

The Shannon diversity index *H* was calculated to characterize bacterial diversity in each group. *P* values were generally used to indicate significance in univariate analysis. The relationship between the microbiota of the HC and EN subjects was explored using an orthogonal partial least square method with discriminant analysis (OPLS-DA), and principal component analysis (PCA) using SIMCA 14.0 (Umetrics, Stockholm, Sweden). OPLS-DA is a multivariate method used to eliminate the genera related to differences between groups. An OPLS-DA model enhances predictive ability and simplifies interpretation^9^. Following construction of the OPLS model, the variable importance in the projection (VIP) of each genus was calculated to select candidate genera that reflected the difference between the subject groups. A VIP score higher than 1 is commonly utilized in multivariate analysis as the criterion for an important variable for driving the observed group separation^33^. However, it is often difficult to determine such a difference based solely upon VIP values, since VIP >1 only implies that the variable contributes more than average to the model. A p(corr) value is an alternative and complementary parameter^9^. Therefore, extended statistical analysis was performed to generate an S-plot using p(corr) as the *y*-axis to seek out the genera contributing most to the compositions of the oral microbiota in the 2 groups in this study.

### Data Availability

The datasets generated and analysed during the current study are available from the corresponding author on reasonable request.

## Electronic supplementary material

Dataset 1
