# Supplementary material for: Composition of salivary microbiota in elderly subjects
Source: Sci Rep. 2018 Jan 11;8:414. doi: 10.1038/s41598-017-18677-0 (PMC5765146; doi:10.1038/s41598-017-18677-0)
Supplement: Supplementary file 1 — Dataset 1 [file 41598_2017_18677_MOESM1_ESM.docx]

**Composition of salivary microbiota in elderly subjects**

Taiji Ogawa^1*^, Yujiro Hirose^2^, Mariko Honda-Ogawa^2^, Minami Sugimoto^3^, Satoshi Sasaki^3^, Masahito Kibi^1^, Shigetada Kawabata^2^, Kazunori Ikebe^1^, Yoshinobu Maeda^1^

^1^Department of Prosthodontics, Gerodontology and Oral Rehabilitation, Osaka University Graduate School of Dentistry, 1-8, Yamadaoka, Suita, Osaka, 5650871, Japan

^2^Department of Oral and Molecular Microbiology, Osaka University Graduate School of Dentistry, 1-8, Yamadaoka, Suita, Osaka, 565-0871, Japan

^3^Department of Social and Preventive Epidemiology, School of Public Health, The University of Tokyo, 7-3-1 Hongo, Bunkyo, Tokyo, 1130033, Japan

**Correspondence to:** Taiji Ogawa

Department of Prosthodontics, Gerodontology and Oral Rehabilitation, Osaka University Graduate School of Dentistry, 1-8, Yamadaoka, Suita, Osaka, 565-0871, Japan

Tel: (+81)-6-6879-2956, Fax: (+81)-6-6879-2957

Email: t-ogawa@dent.osaka-u.ac.jp

**Dietary assessment**

Two research dietitians recorded all food and drink consumed by the EN group subjects on 7 consecutive survey days. The three nursing homes participated to this study provided all meals to their residents. The menu provided was basically same in each facility but the ingredients, amount, and hardness of the meals were arranged according to the individual physical condition including swallowing ability. Each prepared dish after cooking and all drinks were weighed before consumption, then those left as unconsumed were weighed after the subjects had finished the meal, and the amounts of consumed ingredients and seasonings were calculated. Research dietitians assigned food item numbers to all recorded foods and beverages according to the Standard Tables of Food Composition in Japan, Fifth Revised and Enlarged Edition (http://www.mext.go.jp/b_menu/shingi/gijyutu/gijyutu3/toushin/05031802.htm). All collected records were checked by trained dietitian staff at the survey center.

Dietary assessment of the HC subjects was performed using a structured questionnaire, the brief-type self-administered diet history questionnaire (BDHQ), regarding the frequency of consumption of selected foods commonly used in Japanese cuisine, general dietary behavior, and usual cooking methods, as previously described^34^. Briefly, BDHQ is a 4-page fixed-portion questionnaire that asks about the consumption frequency of selected foods, but not about portion size, to estimate the dietary intake of 58 food and beverage items during the preceding month^1^. The BDHQ consists of 5 sections: (1) intake frequency of food and nonalcoholic beverage items, (2) daily intake of rice and miso soup, (3) frequency of drinking and amount per drink for alcoholic beverages, (4) usual cooking methods, and (5) general dietary behavior. BDHQ had been validated for use in assessing 42 selected nutrients intake of Japanese.

The obtained data was adjusted by total energy using the density method, which involved computing the amount of each food group consumed daily per 1000 kcal of daily energy intake for non-energy-providing nutrients. Energy-providing nutrients were expressed as a percentage from daily energy. Validation of the density method was previously established^2^.

**References**

1. Kobayashi, S. *et al.* Both comprehensive and brief self-administered diet history questionnaires satisfactorily rank nutrient intakes in Japanese adults. *J Epidemiol* **22,** 151-159, (2012).

2. Sasaki, S., Yanagibori, R. & Amano, K. Self-administered diet history questionnaire developed for health education: a relative validation of the test-version by comparison with 3-day diet record in women. *J Epidemiol* **8,** 203-215, (1998).

**Table S1. Medical profile of the EN participants**

| **# Age (M/F) Nursing facility Diseases and disorders Care levels ?** |
| --- |
| Subject 1 90 (F) A Dementia 2 |
| Subject 2 68 (F) A Traumatic axonal injury 5  Traumatic brain injury  Presenile dementia  Higher brain dysfunction |
| Subject 3 88 (F) A Congestive heart failure 3 |
| Subject 4 74 (F) B *Data unavailable 1 |
| Subject 5 90 (F) B *Data unavailable 1 |
| Subject 6 81 (F) C Diabetes 2  Right femoral neck fracture  The upper right humerus condylar fracture  Right elbow contracture |
| Subject 7 85 (M) C Diabetes 3  Hypertension  Left femoral neck fracture |
| Subject 8 83 (F) C Hypertension 3  Pyogenic spondylitis  Pyelonephritis |
| Subject 9 82 (M) C Hypertension 2  Hypothyroidism  Thoracic vertebrae (12^th^) compression fracture |
| Subject 10 88 (F) C Dementia 1  Hyperlipidemia,  Hepatitis C |
| Subject 11 77 (F) C Cerebral infarction 2  Hypertension |
| Subject 12 87 (F) C Alzheimer's type dementia 2  Hypertension  Heart failure  Lumbar spinal canal stenosis |
| Subject 13 101 (F) C Hypertension 3  Atrial Fibrillation  Old cerebral infarction  Degenerative lumbar spine disease  Osteoporosis, Rheumatoid arthritis |
| Subject 14 83 (F) C Urinary tract infection 5  Sepsis  Rheumatoid arthritis  Hypertension |
| Subject 15 86 (M) C Chronic renal failure 4  Renal sclerosis  Old myocardial infarction  Paroxysmal atrial fibrillation  Hypertension  Hearing loss  Abdominal dissociative aortic aneurysm  Superior mesenteric artery stenosis  Arteriosclerosis obliterans  Chronic heart failure |

**Table S2. Dietary assessment profile of the participants**

| Elderly in nursing home (EN) Independent living healthy control (HC) |
| --- |
| Nutrient and energy intake (mean)^a^  Total energy [kcal] 1594 1922  Carbohydrates [%energy] 57.5 55.7  Proteins [%energy] 14.3 16.0  Fats [%energy] 26.5 27.0  n-3 polyunsaturated fat [%energy] 1.29 1.30  n-6 polyunsaturated fat [%energy] 5.26 4.81  Sodium [mg/1000 kcal] 2074 2321  Potassium [mg/1000 kcal] 1362 1576  Calcium [mg/1000 kcal] 297 353  Retinol [mg/1000 kcal] 84.9 227  Vitamin B6 (pyridoxine) [mg/1000 kcal] 0. 93 0.88  Vitamin B12 (cobalamins) [µg/1000 kcal] 3.33 5.50  Vitamin C [mg/1000 kcal] 46.9 86.6  Vitamin D [µg/1000 kcal] 4.53 8.50  Total dietary fiber [g/1000 kcal] 7.00 7.88  Water [g/1000 kcal] 942 973 |

**^a^** Nutrient and energy intakes were assessed with 7-day diet records among EN group and with brief-type self-administered diet history questionnaire among HC group. Energy adjustment was performed according to the density method.
